# Beyond the chimeric antigen receptor T cells and bispecific antibody duopoly: ex vivo armed T cells for solid tumors

**DOI:** 10.3389/fimmu.2026.1822523

**Published:** 2026-05-26

**Authors:** Jeong A Park, Nai-Kong V. Cheung

**Affiliations:** 1 Department of Pediatrics, Inha University Hospital, Inha University College of Medicine, Incheon, Republic of Korea; 2 Department of Pediatrics, Memorial Sloan Kettering Cancer Center, New York, NY, United States

**Keywords:** bispecific antibody, chimeric antigen receptor T cell, *ex vivo* armed T cell, multi-antigen targeting strategy, on-target off-tumor toxicity, T cell immunotherapy, tumor heterogeneity, tumor microenvironment

## Abstract

While monoclonal antibodies (mAbs) continue to dominate the overall immunotherapy landscape, the field of T-cell-based therapeutics is rapidly evolving. Although chimeric antigen receptor T cells (CAR-T) and bispecific antibodies (BsAbs) currently represent the pillars of T-cell-directed therapy, the complexity of solid tumors demands a more diversified therapeutic arsenal. By combining antibody-mediated tumor targeting with the robust effector function of ex vivo expanded T cells, BsAb-armed T cells (BATs)-also referred to as Ex vivo Armed T cell (EATs)-provide a ‘third way’ that addresses the unmet needs of solid tumor immunotherapy. They can overcome the quantitative and qualitative deficiencies of endogenous immune effector cells in cancer patients. By offering personalized multi-antigen targetability and the prospect of off-the-shelf therapy, EATs have the potential to address critical challenges, such as poor tumor infiltration, immune escape via heterogeneity and target antigen loss, and treatment-related toxicities like cytokine release syndrome. In this review, we discuss the characteristics of EAT therapy, distinct from CAR-T and BsAb therapy, as an independent and alternative niche. We explore strategies to accelerate their clinical translation, encompassing BsAb optimization, modulation of the tumor microenvironment (TME) and cytokines, and simultaneous engagement of multiple antigens, which are essential for boosting EAT potency and overcoming the limitations of solid tumors. In this evolving landscape, EATs could play a unique and independent role, expanding the CAR-T and BsAb-dominated paradigm to address unmet clinical needs.

## 1 Introduction

Cancer immunotherapy has now revolutionized the field of oncology by prolonging survival of patients with previously fatal cancers. Immunotherapy holds the potential to be more precise, more personalized, more effective, and less toxic than current chemotherapies. The number of patients eligible for immune-based cancer programs continues to rise as these therapies are integrated into standard care. Novel treatment combinations and newly identified targets further expand the scope and clinical stage of malignancies in which immunotherapy could provide a significant survival benefit. Among them, T cell immunotherapy using chimeric antigen receptors (CARs) or bispecific antibodies (BsAbs) bypassing the MHC hurdles has shown considerable promise. Yet, the success in solid tumors has lagged behind compared to those in hematologic malignancies, and by extension, the clinical impact of its application in solid tumors has remained modest. The complexity of the solid tumor microenvironment (TME) suggests that no single ‘panacea’ can solve every clinical challenge. Just as a diverse ecosystem is more resilient, the immunotherapy landscape requires a multimodal approach. Here, we undertake a comprehensive review, focusing on how the EAT approach addresses the specific challenges encountered by CAR-T cells (CAR-T) and BsAb therapies in solid tumors. As a unique and independent niche within the broadening immunotherapy landscape, we highlight the promises of Ex vivo Armed T cells (EATs; also referred to as BsAb-armed T cells, BATs) and explore strategies to accelerate their clinical translation.

## 2 Challenges in CAR-T therapy

CAR-T therapy has achieved remarkable success in the treatment of hematologic malignancies (1), and seven CAR-T products have been approved by the US FDA (2). However, CAR-T therapy requires a resource-intensive process (3) and continues to face significant efficacy hurdles in the treatment of solid tumors. Attempts to translate CAR-T therapy to solid tumors have yielded disappointing clinical results and revealed key challenges that constrain therapeutic efficacy, including the limited ability of CAR-T to traffic to and infiltrate the tumors (4). The immune-hostile TME, characterized by physical barriers, dysregulated microvasculature, hypoxia, acidity, and immunosuppressive tumor-infiltrating leukocytes, such as myeloid-derived suppressor cells (MDSCs), tumor-associated macrophages (TAMs), and regulatory T cells (Tregs), remains a formidable obstacle, hindering CAR-T’s access, activity, and effectiveness even with intra-tumoral injections (5, 6). Furthermore, tumors evolve under the constant pressure of target antigen-specific CAR-T by downregulating their target antigens, thereby evading CAR-T recognition (7).

While sustained CAR-T presence is often associated with durable responses in CD19(+) B-cell malignancies, this long-term persistence frequently leads to chronic B-cell aplasia. Although B-cell aplasia serves as a clinical indicator of *in vivo* CAR-T activity, it necessitates long-term immunoglobulin replacement, imposing a significant burden on patients (8). Furthermore, the reliance on long-term persistence for efficacy is a double-edged sword; whereas curtailed persistence increases the risk of disease relapse, prolonged persistence not only leads to chronic immune deficiency but may also provide the selective pressure for antigen-negative relapse (9). Furthermore, the potential for secondary T-cell malignancies remains a critical safety consideration that warrants lifelong monitoring for recipients. Following the U.S. FDA’s report of 22 cases (10), secondary T-cell lymphoma has been identified as a potential long-term complication (11, 12). The emergence of CAR-expressing T-cell lymphomas highlights the risk of insertional mutagenesis, where vector integration disrupts host gene expression and facilitates malignant transformation (12, 13), underscoring the inherent safety challenges of genetically modified therapies and the need for vigilant longitudinal monitoring.

## 3 Challenges in BsAb therapy

The other pillar of the duopoly, BsAb, has emerged as a promising strategy by simultaneously targeting tumor antigens and T or NK cells in an FcγR- and MHC-independent manner. Following successful clinical development and FDA approval in B cell malignancies, the focus of BsAb therapies has rapidly expanded toward numerous candidates targeting solid tumors (**Table 1**). As IgG-like engineered antibodies, BsAbs facilitate a streamlined clinical workflow and ‘off-the-shelf’ accessibility. The risks of severe cytokine release syndrome (CRS) or immune effector cell-associated neurotoxicity syndrome (ICANS) are significantly lower than those associated with CAR-T (14, 15), and the half-lives of BsAbs, ranging from 2.1 hours to 21 days, offer predictable pharmacokinetics, in contrast to the potentially life-long persistence of CAR-T (16). Besides, BsAb can be logically combined with other therapeutic modalities, such as chemotherapy, immune checkpoint inhibitors (ICIs), and other targeted therapies, to enhance therapeutic responses through complementary mechanisms of action (16). However, because BsAbs rely on endogenous effector T cells for their activity, their anti-tumor efficacy may be limited in patients with impaired or insufficient immune effector cells. Furthermore, the rapid systemic clearance of BsAbs typically necessitates continuous infusion or frequent administration to maintain therapeutic levels. While increasing the dose could theoretically compensate for this rapid clearance, the risk of treatment-related toxicities, though lower than that of CAR-T, often prevents the dose-escalation required to overcome the immunosuppressive TME (17).

**Table 1:** Global approved bispecific antibodies.

Generic name	Trade name	Approval	Target(s)	Format	Engineering/platform	Mechanism of action	Indication
Blinatumomab	Blincyto^®^	2014 (FDA)	CD19 and CD3	BiTE (scFv-based)	BiTE	T-cell engager targeting CD19(+) B cells	Relapsed or refractory B cell precursor ALL
Emicizumab	Hemlibra^®^	2017 (FDA)	Factor IXa and Factor VIII	Asymmetric IgG4	CrossMab + Knobs-into-Holes	Mimics FVIII cofactor bridging FIXa and FX	Hemophilia A
Amivantamab	Rybrevant^®^	2021 (FDA)	EGFR and cMet	Bispecific IgG1	DuoBody	Dual targeting of EGFR and MET	Locally advanced or metastatic NSCLC, EGFR exon 20 mutated
Faricimab	Vabysmo^®^	2022 (FDA)	VEGF-A and Ang-2	Bispecific IgG1	CrossMab	Dual inhibition of angiogenesis pathways	Exudative age-related macular degeneration and diabetic macular edema
Tebentafusp	Kimmtrak^®^	2022 (FDA)	GP100 and CD3	Bispecific TCR-mimic fusion	TCR bispecific fusion (Immunocore)	Redirects T cells to melanoma cells expressing gp100 peptide-HLA-A*02:01	Unresectable or metastatic uveal melanoma
Teclistamab	Tecvayli^®^	2022 (FDA)	BCMA and CD3	Bispecific IgG4	DuoBody	T-cell engager targeting BCMA(+) myeloma cells	Relapsed or refractory MM
Elranatamab	Elrexfio^®^	2023 (FDA)	BCMA and CD3	Bispecific IgG2a	Modified IgG (Hinge mutation in CH3 domains)	T-cell engager targeting BCMA(+) myeloma cells	Relapsed or refractory MM
Epcoritamab	Epkinly^®^	2023 (FDA)	CD20 and CD3	Full-length bispecific IgG1	DuoBody	T-cell engager targeting CD20(+) B cells	Relapsed or refractory DLBL and high-grade B-cell lymphoma
Glofitamab	Columvi^®^	2023 (FDA)	CD20 and CD3	Full-length bispecific IgG1	CrossMab	T-cell engager (2:1 ratio) targeting CD20(+) B cells	Relapsed or refractory DLBL
Mosunetuzumab	Lunsumio^®^	2023 (FDA)	CD20 and CD3	Bispecific IgG1	Knobs-into-holes	T-cell engager targeting CD20(+) B cells	Relapsed or refractory FL
Talquetamab	Talvey^®^	2023 (FDA)	GPRC5D and CD3	Bispecific IgG4	Duobody	T-cell engager targeting MM	Relapsed or refractory MM
Cadonilimab	Kaltanni^®^	2023 (NMPA)	PD-1 and CTLA-4	Bispecific IgG1	Tetrabody	Dual checkpoint inhibition	Relapsed or refractory cervix cancer
Zanidatamab	Ziihera^®^	2024 (FDA)	2 HER2 domains (ECD2 and ECD4)	Biparatopic IgG1	Azymetric™ platform	Dual HER2 blockade via receptor clustering and internalization	HER2-positive biliary tract cancer
Tarlatamab	Imdelltra^®^	2024 (FDA)	DLL3 and CD3	BiTE-Fc (scFv based)	BiTE-Fc	T-cell engager targeting DLL3 (+) lung cancer cells	Extensive stage SCLC
linvoseltamab-gcpt	Lynozyfic^®^	2025 (FDA)	BCMA and CD3	Full-length, bispecific IgG4	Veloci-Bi^®^	T-cell engager targeting BCMA(+) myeloma cells	Relapsed or refractory MM

ALL, acute lymphoblastic leukemia; Ang2, angiopoietin-2; Azymetric™: Zymeworks’s next-gen knobs-into-holes + scFv/Fab fusion; BCMA, B cell maturation antigen; BiTE, bispecific T cell engager; c-MET, cellular mesenchymal epithelial transition; CTLA-4, cytotoxic T lymphocyte-associated protein 4; DLBL, diffuse large B cell lymphoma; DLL3, delta-like ligand 3; DuoBody^®^, Genmab’s controlled Fab-arm exchange; ECD, extracellular domain; EGFR, epidermal growth factor receptor; FL, follocular lymphoma; GP100, glycoprotein 100; GPRC5D, G protein-coupled receptor class C group 5 member D; HER2, human epidermal growth factor receptor 2; MM, multiple myeloma; NSCLC, non-small cell lung carcinoma; PD-1, programmed cell death protein 1; SCLC, small cell lung cancer; TNF-α, tumor necrosis factor-α; TCR, T cell receptor; VEGF-A, vascular endothelial growth factor-A; Veloci-Bi^®^, Regeneron native pairing full-IgG.

Concerning therapeutic access to the central nervous system (CNS), theoretically, BsAbs can penetrate the CNS, though only when the blood-brain barrier (BBB) is disrupted (18). In malignancies where the BBB remains intact, CNS relapse persists a major cause of treatment failure (19). Notably, however, the BBB is frequently impaired in CNS malignancies due to tumor-induced neoangiogenesis and inflammation. While this impairment provides a potential therapeutic window for BsAb therapy, as evidenced by the use of EGFRvIII-targeted T cell engagers in glioblastoma (20, 21), significant challenges remain. Achieving therapeutic concentrations within the CNS via systemic administration requires high serum levels to overcome the BBB and the physical barriers of solid tumors. This pharmacological requirement, combined with rapid antibody clearance, necessitates high-intensity dosing schedules that consequently elevate the risk of systemic toxicities.

## 4 *Ex vivo* armed T cell therapy

**Figure 1:**
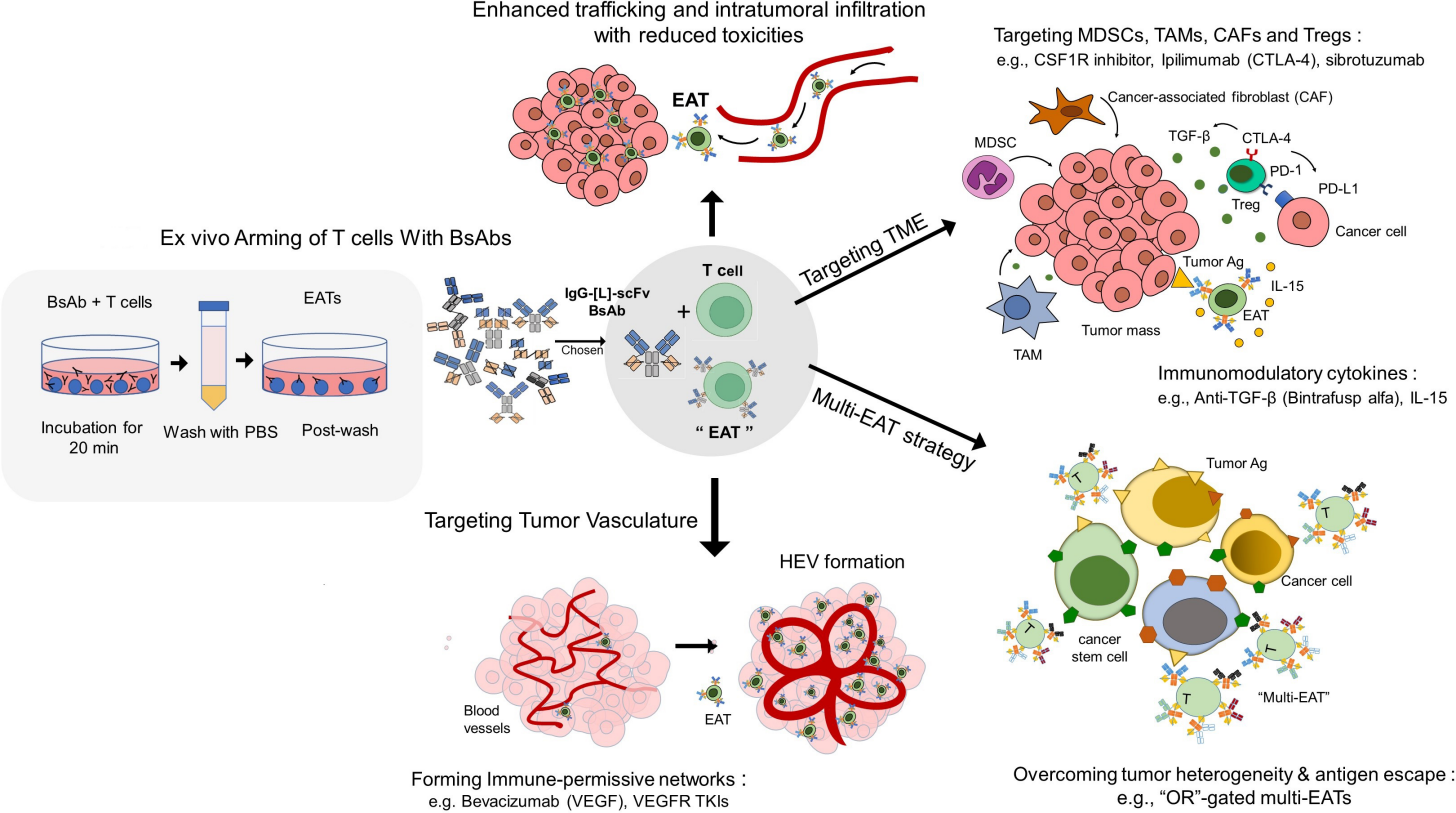
Strategies for enhancing the efficacy of EAT therapy. When ex vivo-expanded T cells are armed with IgG-[L]-scFv formatted BsAbs, these EATs (Ex vivo Armed T cells) demonstrate enhanced tumor infiltration along with significantly reduced cytokine release. The efficacy of EAT therapy can be further optimized through several integrated strategies (1): Modulating the tumor microenvironment (TME) by depleting or reprogramming immunosuppressive cells, such as myeloid-derived suppressor cells (MDSCs), tumor-associated macrophages (TAMs), and regulatory T cells (Tregs) (2); Targeting immunomodulatory cytokines, including the neutralization of inhibitory factors like TGF-β or the leveraging of stimulatory cytokines such as IL-2 and IL-15 to enhance T-cell fitness; and (3) Targeting tumor microvasculature via vascular endothelial growth factor (VEGF) inhibition. Anti-VEGF antibodies induce the formation of high endothelial venules (HEVs) and transform the hypoxic TME into an immunopermissive environment, thereby facilitating EAT dispersion. Additionally, EAT therapy can overcome inherent resistance mechanisms- including tumor heterogeneity and antigen escape- through the simultaneous targeting of multiple tumor antigens and cancer stem cells (CSCs). Collectively, these approaches enable EAT therapy to more effectively transform “cold” tumors into “hot” tumors, demonstrating significant potential for clinical synergy.

To address the limitations of these two modalities, Ex vivo Armed T cells (EATs) offer a viable strategy that bridges the cellular potency of CAR-T with the versatile targeting of the BsAbs (**Table 2**). EATs use T cells as active delivery vehicles, and the armed BsAbs serve as a molecular guide, directing T cell infiltration into the tumor and anchoring the targeting moiety within the TME. The EAT platform prevents the rapid systemic washout of the antibodies and enables a high local concentration of T cells at the tumor site, potentially circumvent the need for continuous, high-dose systemic administration (22, 23). EATs are generated by a three-step process (**Figure 1**). First, T cells are expanded *ex vivo* to ensure a high-quality effector population. Second, these T cells are ‘armed’ by incubating them with T-BsAbs, which coat the T cell surface via the anti-CD3 moiety. Finally, these armed T cells are washed to remove released cytokines and unarmed BsAbs to reduce unwanted infusion-related toxicities. Upon infusion, the tethered BsAbs drive T cell extravasation and infiltration into the tumor parenchyma via specific engagement with TAAs. This engagement facilitates the formation of a stable immunological synapse, triggering T-cell degranulation and the localized release of perforin and granzymes, ultimately leading to tumor cell lysis (24). By adopting the ex vivo expansion method, EATs circumvent the limitations of insufficient and/or dysfunctional patient-derived T cells while mitigating the short half-lives typically associated with BsAb therapy. Similar to CAR-T, EATs circumvents the classic HLA restriction by recognizing surface antigens on a tumor cell, but unlike CAR-T armed with a gene introduced by a viral vector (to code for the scFv of CAR), EAT is armed with a protein, ‘off-the-shelf’ T-BsAb (24). EAT remains inactive until its contact with tumor target, which initiates a rapid cascade of downstream signals, leading to T cell activation and tumor cell lysis. While cytotoxicity of CAR-T requires two signals activating two separate downstream cascades, i.e. CD3 and 41BB or CD28, EAT engages a single pathway, CD3 without additional co-stimulatory activation, thereby avoiding both over-activation and early exhaustion of T cells. In contrast to CAR-T, the more transient pharmacokinetic profile of EATs—which peak in tumors between days 7 and 14 and persist for approximately 30 days—offers a predictable therapeutic window that may mitigate the risks associated with lifelong effector cell presence (24). In addition, since nonspecific cytokines are removed before infusion or storage, significantly less cytokine release was seen following EAT therapy (24, 25). Compared to direct BsAb injection, EATs showed more rapid intratumoral infiltration, with substantially reduced T helper I (TH1) cell cytokines, particularly TNF-α, which should result in lower CRS and neurotoxicity (24). When attached through CD3 to T cells, the T-BsAb exhibits reduced clearance compared to its stand-alone form, which should translate into more durable anti-tumor responses (26, 27).

**Table 2:** Comparative features of T-BsAbs, CAR-T, and EATs in solid tumor immunotherapy.

Category	Dimension	CD3(+) T-BsAbs	CAR-T	EATs/BATs
Mechanism	Effector	Endogenous T cells via CD3.	Autologous T cells engineered with a CAR.	Autologous polyclonal T cells pre-armed ex vivo.
	Antigen recognition	Mostly surface antigens; HLA-independent or ImmTACs; HLA-restricted.	Surface antigens; HLA-independent.	Surface antigens; HLA-independent
	Mode of action	Synthetic immune synapse (CD3–tumor antigen) induces T cell activation and serial killing.	Stable CAR expression enables persistent antigen recognition and killing.	Synthetic immune synapse via pre-armed coat; no intrinsic costimulation.
	Antigen breadth & adaptability	Single-antigen focused; limited adaptability for new targets.	Fixed specificity; require new viral engineering for dual-targeting.	Highly modular; supports multi-antigen targeting (multi-EATs).
Efficacy	Potency	Moderate–high; Reliant on the quantify and fitness of endogenous T cells.	Very high; Potent but prone to exhaustion.	High; driven by optimized, pre-expanded effector cells.
	Persistence	Short (hours–days); needs step-up/continuous exposure.	Long (months–years); active *in vivo* expansion.	Intermediate (days–weeks); requires re-dosing; finite lifespan as coat wanes.
	Clinical activity	Proof-of-concept approvals; tebentafusp in uveal melanoma; tarlatamab in SCLC	Broad early trials; first registration programs (CLDN18.2)	Early-phase signals; feasible, safe
Safety	CRS	Intermediate–high; Systemic activation	High; Driven by rapid *in vivo* expansion	Low–intermediate; Limited by pre-infusion cytokine wash.
	Neurotoxicity (ICANS)	Low-intermediate	High	Low
	Class-specific adverse effects	CRS/ICANS on label for several agents	CRS/ICANS, prolonged cytopenia, hypogammaglobinemia	Mostly grade 1–2 flu-like symptoms, no high-grade CRS/ICANS reported in trials.
	On-target off-tumor toxicity	Risk if antigen expressed on normal tissues	Highest risk due to potential for indefinite cell persistence.	Target-dependent; milder due to finite therapeutic window.
Practicality	Manufacturing	Off-the-shelf	Autologous collection, engineering, expansion	Autologous T-cell isolation + arming of off-the-shelf BsAbs
	Administration	IV infusion or SC, Step-up dosing, Sometimes continuous IV	IV infusion; single-dose cell therapy	IV infusion; repeated cycles (weekly or biweekly)
	Conditioning	No lymphodepletion	Requires lympho-depleting chemotherapies (Flu/Cy) 3–5 days prior to CAR-T therapy	No lymphodepletion
	logistic burden	Low; requires step-up dosing, CRS monitoring- inpatient or outpatient clinic	Very high; centralized manufacture; inpatient monitoring	Intermediate-high; repeated outpatient cell infusions; requires autologous cell processing.
Clinical maturity	Clinical footprint	Tebentafusp (gp100–HLA-A*02:01 × CD3); Tarlatamab (DLL3×CD3).	No approvals yet; CLDN18.2 CAR-T (satri-cel) under review in China.	Early-phase clinical trials; translational/clinical reports in solid tumors are emerging.
	Strength	High potency via T-cell redirection; demonstrated clinical efficacy.	Potent/durable if TME barriers overcome; armored/tandem/regional CAR-T are promising.	T-cell redirection without engineering; repeat dosing possible, multi-antigen targetability.
	Limitations	Dependence on endogenous T cells.	Manufacturing burden; Risk of secondary malignancies.	Requires cell processing infrastructure.; Activity wanes as coat is lost.

BATs, bispecific antibody-armed T cells; BsAb, bispecific antibody; CAR-T, chimeric antigen receptor T cells; CLDN18.2, Claudin 18.2 (tight junction protein); CRS, cytokine release syndrome; DLL, delta-like ligand 3; EATs, Ex vivo Armed T cells with bispecific antibody; EGFR, epidermal growth factor receptor; Flu/Cy, fludarabine and cyclophosphamide; gp100, glycoprotein 100; HLA, human leukocyte antigen; ICANS, immune effector cell–associated neurotoxicity syndrome; ImmTACs, immune-mobilizing monoclonal TCRs against cancer; IV, intravenous; NSCLC, non–small cell lung cancer; PD-L1, programmed death-ligand 1; SC, subcutaneous.

In clinical trials, EATs (BATs) were safe at cell doses as high as 8x10^10^ and have demonstrated clinical activity across a range of malignancies, including various solid tumors (28–30). Target-specific EATs successfully infiltrated and regressed tumors while stimulating memory T cell subsets. These subsets established a robust anti-tumor immunity capable of resisting subsequent tumor rechallenge (31). The clinical trials of EATs are summarized in **Table 3**. Anti-GD2 EATs demonstrated a favorable safety profile and exhibited clinical activity with anti-GD2 immunity in patients with recurrent/refractory neuroblastoma (NB) and osteosarcoma (32). Anti-CD20 EATs following high-dose chemotherapy with autologous hematopoietic stem cell transplantation (HCT) also induced humoral and cellular tumor-specific immunity without impairing engraftment or antibody recovery in patients with non-Hodgkin lymphoma (NHL) and multiple myeloma (33, 34). While several early-phase trials of EATs/BATs utilized concomitant low-dose IL-2 or GM-CSF, these agents are primarily administered as supportive measures to ensure the *in vivo* survival and expansion of the infused cells. Given that low-dose cytokine monotherapy rarely yields objective responses in advanced solid tumors, the observed clinical activity is likely attributable to the EAT product itself. Beyond these supportive regimens, more intensive combinations—including ICIs, chemotherapy, and radiotherapy—are being actively investigated to enhance clinical outcomes for patients with high-risk disease (22, 29). The combination of pembrolizumab and anti-HER2 EATs was well tolerated and demonstrated clinical efficacy in metastatic castration-resistant prostate cancer (CRPC) (29), underscoring the potential synergy between EATs and other therapeutic modalities. However, these combination regimens may complicate the interpretation of the independent efficacy of EAT therapy.

**Table 3:** Clinical trials of *ex vivo* armed T cells.

Clinical trial	Target	Eligibility (years)	Phase	Status	Concurrent therapy	ORR (%)	NCT number
High-dose chemotherapy followed by autologous HCT and CD20-EATs for patients with NHL	CD20 x CD3	≥ 15	Phase 1	Completed	High-dose chemotherapy plus autologous PBSCT	NA	NCT00244946
CD20-BATs followed by autologous HCT in treating patients with MM (34)	CD20 x CD3	≥ 18	Phase 1	Completed	High-dose chemotherapy plus autologous PBSCT	NA	NCT00938626
HER2-BATs for HER2-negative metastatic breast cancer.	HER2 x CD3	≥ 18	Phase 2	Completed	IL-2 plus GM-CSF	ORR 46.8%; OS, 13.8 mo.	NCT01022138
EGFR-EATs for advanced colorectal or pancreatic cancer (30)	EGFR x CD3	≥ 18	Phase I	Completed	IL-2 plus GM-CSF	CR (10%), SD (20%), OS, 21.1 mo.	NCT01420874
	EGFR x CD3	≥ 18	Phase 2	Completed			NCT02620865
	EGFR x CD3	≥ 18	Phase 2	Active, not recruiting			NCT03269526
GD2-BATs in children and young adults with neuroblastoma and osteosarcoma (32)	GD2 x CD3	1.1 - 29	Phase 1 & 2	status varied	IL-2 plus GM-CSF	OS, 21.1 mo.; CR (8.3%), PR (8.3%), SD (25%)	NCT02173093
HER2-EATs for HER2 (+) esophageal cancer, gastric cancer, pancreatic cancer, liver or GB cancer, or colon cancer	HER2 x CD3	≥ 18	Phase 1	Unknown status	IL-2	NA	NCT02662348
Activated CIK-MUC1/CEA/EpCAM/GPC3- EATs for advanced liver cancer	MUC1 x CD3, CEA x CD3, EpCAM x CD3, and GPC3 x CD3	>18	Phase 2	Unknown status		PFS, 4 mo.; OS,13.2 mo.; DCR, 63.6%	NCT03146637
HER2-BATS and pembrolizumab in metastatic breast cancer	HER2 x CD3	≥ 18	Phase 1 & 2	Active, not recruiting	Pembrolizumab	NA	NCT03272334
EGFR-BATs in combination with temozolomide and radiation in patients with glioblastoma (GBM) (22)	EGFR x CD3	≥ 18	Phase 1	completed	Temozolomide plus radiotherapy	PFS,17.2 mo.; OS, 28.8 mo.	NCT03344250
Pembrolizumab and HER2-BATs in treating patients with metastatic castration resistant prostate cancer (CRPC) (29)	HER2 x CD3	≥ 19	Phase 2	Completed	Pembrolizumab	PFS, 5 mo.; OS 31.6 mo.; 6 mo. PFS, 38.5%	NCT03406858
EGFR-EATs in patients with advanced pancreatic cancer	EGFR x CD3	≥ 18	Phase 1	Active, not recruiting		NA	NCT04137536
CD30-BATs for CD30 (+) malignant lymphoma/leukemia	CD30 x CD3	≥ 18	Phase 1	Recruiting	GM-CSF	NA	NCT05544968
EGFR-BsAb-armed PBMCs in metastatic or unresectable pancreatic cancer	EGFR x CD3	≥ 18	Phase 1 & 2	Recruiting		NA	NCT06479239

BATs, Bispecific antibody Armed T cells; CEA, carcinoembryonic antigen; CIK, Cytokine-Induced Killer cells; CR, complete response; CRPC, castration resistant prostate cancer; DCR, disease control rate; EATs, Ex vivo Armed T cells; EGFR, *Epidermal Growth Factor Receptor;* EpCAM, Epithelial Cell Adhesion Molecule; GB, gallbladder; GBM, Glioblastoma Multiforme; GPC3, Glypican-3; HCT, Hematopoietic Cell Transplantation; HER2/Neu, *Human Epidermal Growth Factor Receptor 2* (ERBB2, also called Neu in rodents); MM, multiple myeloma; mo., months; MUC1, Mucin-1; NA, not available; NHL, Non-Hodgkin lymphoma; ORR, overall response rate (≥SD); OS, overall survival; PBMC, peripheral blood mononuclear cell; PBSCT, peripheral blood stem cell transplantation; PFS, progression-free survival; PR, partial response; SD, stable disease, yr, years

## 5 Enhancing the efficacy of EAT therapy for solid tumors

### 5.1 Enhancing BsAb potency and efficacy

While early results are promising, further optimization of BsAb design is required to improve EAT efficacy and consistency in solid tumors. Structural format critically influences T cell trafficking and cytotoxicity. Initial approaches using chemically conjugated anti-GD2/anti-CD3 IgGs demonstrated feasibility (35), but smaller formats such as tandem scFvs (BiTEs) improved tumor penetration and T cell infiltration (36). Enhancing tumor antigen affinity markedly increased potency (37–39), while CD3 affinity required careful tuning: higher CD3 or target antigen affinity induced severe cytokine surge and toxicity, whereas lower CD3 affinity improved *in vivo* efficacy despite reduced *in vitro* potency (40–42). These findings highlight the importance of balancing affinities for both CD3 and the target antigen to optimize the therapeutic index, which is crucial for improving the therapeutic index of T-BsAbs.

However, monomeric BiTEs (tandem scFvs) have limitations, including short half-life and suboptimal pharmacokinetics, necessitating continuous infusion (43), Fc-engineered formats and half-life-extended BiTEs partially address these issues. More advanced designs, such as the 2:1 CD20xCD3 BsAb (e.g., glofitamab) and half-life-extended BiTEs (e.g., tarlatamab), enhance tumor avidity while limiting CD3 overactivation, thereby improving both efficacy and safety (44–46). Notably, the IgG-based tetravalent formats (IgG-[L]-scFv) further improves pharmacokinetics and tumor delivery compared to monomeric BiTE while maintaining controlled CD3 engagement (47). The molecular size of IgG-[L]-scFv (210 kDa) remains within the Goldilocks zone, balancing between sufficiently slow blood clearance and effective vascular extravasation for T cell delivery (48). This format has demonstrated robust T cell infiltration and antitumor activity compared with other BsAb structures and proven particularly effective for generating EATs, underscoring the critical role of BsAb format in optimizing therapeutic outcomes (24, 47, 49).

### 5.2 Factors determining *in vivo* efficacy of EATs

To further optimize EAT therapy, the arming density of BsAb was examined in the preclinical settings. EAT potency depended on the BsAb arming dose, with optimal efficacy at intermediate doses (0.05–5 µg/10^6^ T cells). Beyond a certain threshold, increasing the BsAb arming dose did not enhance anti-tumor effects; rather, it increased activation-induced cell death (AICD) markers, reducing cytotoxicity. This highlights the critical necessity of identifying an optimal arming dose to maximize therapeutic benefit (24, 50). The EAT cell dose also contributed to the vivo antitumor effects; the tumor-suppressing effect consistently increased with the number of EATs infused, and a subsequent dose of EATs further enhanced T cell proliferation and sustained tumor control. In addition, while once-a-week schedule of EATs was sufficient to ablate slowly growing tumors, a dose-dense schedule (2 or 3 doses per week) was more effective for large or rapidly growing tumors (24). This implicates that treatment intensity can be readily modulated according to disease status, allowing for tailored dosing (lower or fewer doses for low disease burden, larger or more frequent doses for high disease burden) to optimize treatment outcomes.

### 5.3 Overcoming tumor microenvironment

Tumor heterogeneity, target antigen loss, and the immunosuppressive TME continue to pose major challenges for EAT therapy in solid tumors as well. While these TME targeting strategies are mechanistically orthogonal to EAT therapy and could theoretically benefit other T cell immunotherapies, EATs offer a unique modular scaffold that facilitates the seamless integration of these synergistic agents. The clinical utility of such combinations hinges on the effective delivery of active T cells into the tumor parenchyma; without successful infiltration, the potential synergy of TME-targeting agents remains unexploited. By leveraging the superior trafficking capabilities of the IgG-[L]-scFv platform, EAT therapy ensures robust T cell delivery and allows combination therapies to maximize antitumor efficacy. Strategies for enhancing the efficacy of EAT therapy are illustrated in **Figure 1**. First. targeting TME has emerged to enhance T cell infiltration, particularly in ‘cold’ tumors (51). While immune-inflamed tumors exhibit a robust T-cell immune response driven by IFN-γ signaling, characterized by high PD-L1 expression in tumor cells, abundant tumor-infiltrating lymphocytes (TILs), and intact antigen presentation via HLA and MHC class I, immune-excluded (“cold”) tumors are characterized by TGF-β signaling, which suppresses T-cell infiltration and activity (52). They also share features of a dense and reactive stroma, abundant MDSCs, abnormal tumor angiogenesis, and aberrant activation of Wnt/β-catenin pathway (53, 54). This overactivation fosters an immunosuppressive TME by enhancing glycolysis, glutaminolysis, and lipogenesis to support both bulk tumor cells and cancer stem cells (CSCs) (55). An abundance of MDSCs and TAMs, often accompanied by synthetic immunities, can directly hinder the therapeutic efficacy of T cell immunotherapy by inducing early exhaustion of T cells, promoting inhibitory signaling pathways, and limiting their metabolic support, ultimately contributing to immune evasion and reduced efficacy of immunotherapy (56). To overcome this hostile TME, therapeutic strategies have focused on specific cell types, soluble factors, or both. First, myeloid-directed strategies are proven approaches for improving immunotherapy outcomes in preclinical models. Neutrophils and polymorphonuclear MDSCs can be depleted using anti-Ly6G antibody, monocytes and monocytic MDSCs using anti-Ly6C antibody, or TAMs using anti-CSF1R (colony-stimulating factor 1 receptor) antibody or clodronate liposome. This approach significantly enhanced intratumoral EATs’ infiltration and their proliferation. Notably, depleting TAMs was more effective than targeting specific MDSC subtypes (57). Pan-macrophage depletion significantly enhanced EAT’s anti-tumor efficacy, accompanied by increased infiltration of CD4(+) and CD8(+) T cells, especially in immune-excluded tumors (57), suggesting these tumors harbor predominantly M2-skewed TAMs.

Unexpectedly, corticosteroids improved anti-tumor effect of EAT therapy in a dose-dependent manner (57). While corticosteroids are generally known to be T cell suppressive to varying degrees depending on the specific subset in circulation (58), their effects on monocytes and macrophages depend on the dose and duration of exposure. In the short-term, low-dose exposure promotes monocyte migration into injured tissues, while prolonged, high-dose exposure prohibits monocyte trafficking and impairs macrophage functions via glucocorticoid receptors (59). In preclinical studies, dexamethasone profoundly reduced circulating monocytes in a dose-dependent manner, and high-dose dexamethasone significantly reduced the number of monocytic-MDSCs and macrophages in tumors, allowing more EATs to successfully infiltrate into tumor parenchyma, increasing CD8(+) TILs and enhancing the anti-tumor effect (57).

### 5.4 Therapeutic targeting of regulatory T cells

Targeting Tregs is another compelling strategy that could improve the efficacy of EAT therapy. Tregs often accumulate in tumors, impairing immunosurveillance and anti-tumor responses, which contribute to poor response in patients receiving cancer immunotherapy (60). Tregs exert immunosuppressive effects through multiple mechanisms, including CTLA-4-mediated suppression of antigen presenting cell (APC) maturation, induction of indoleamine 2,3-dioxygenase (IDO), secretion of inhibitory cytokines such as TGF-β, IL-10, and IL-35, extracellular adenosine production, and effector T cell apoptosis (61). Inhibiting Treg activity with ipilimumab (anti-CTLA-4 antibody) during ex vivo T cell expansion could enhance T cell proliferation and boost BsAb-redirected antitumor cytotoxicity of EATs (62). Combination of ipilimumab and anti-EGFR EATs or anti-CD20 EATs also enhanced tumor-specific cytotoxicity against pancreatic cancer and Burkitt’s lymphoma cell lines, through a selective reduction of the Treg population among the expanded T cells, alongside the increased secretion of immunostimulating cytokines, chemokines, and growth factors (63).

### 5.5 Targeting cytokine and chemokine signaling pathways

Cytokines and chemokines are key modulators of the response to cancer immunotherapies, thus targeting these signaling molecules can also serve to enhance the efficacy of EAT therapy. Pro-inflammatory cytokines, such as IL-2, IL-12, IL-15, TNF-α, and IFN-γ, induce cytotoxic T lymphocyte (CTL)-mediated cancer cell death as well as facilitate tumor rejection (64). One of the cytokines with substantial promise is IL-15. Like IL-2, IL-15 promotes T cell proliferation, supports CTL and memory CD8(+) T cell generation, and sustains NK cell expansion and maintenance, but unlike IL-2, IL-15 achieves these effects without increasing Tregs, AICD or vascular leakage syndrome (65). Stable IL-15/IL-15Rα complexes on the surface of APCs induce trans-endosomal recycling of IL-15, driving the generation of high-avidity antigen-specific T cells, an 80- to 100-fold expansion, and the long-term survival of CD8(+) memory T cells (66, 67). IL-15/IL-15Rα Fc-fusion proteins, because of their longer half-lives, further amplified T-BsAb activity enabling complete and durable tumor control (68). These findings underscore the potential of combining IL-15 with EAT therapies- either during incubation or as a co-administration strategy- to enhance EAT survival and therapeutic efficacy.

Conversely, myeloid cells within the TME, along with tumor cells, secrete immunosuppressive cytokines (e.g., IL-10 and TGF-β) to blunt CTL activity (89). The overexpression of VEGF and TGF-β cooperatively suppresses T cell infiltration and activation, contributing to an immune-hostile TME and treatment resistance (69). Neutralizing these protumoral cytokines or blocking their receptors have the potential to modulate the multidimensional ecosystem of the TME and enhance the efficacy of T cell immunotherapies. CD8(+) T cells in the presence of tumor-derived TGF-β fail to activate cytotoxic gene expression including perforin, granzymes, Fas ligand, and IFN-γ, leading to impaired cytotoxicity against cancer cells (70). TGF-β also recruits myeloid cells and facilitates metabolic coupling between cancer and stromal cells by driving cancer-associated fibroblasts to supply energetic metabolites to cancer cells, contributing to treatment resistance (71, 72). Systemic neutralization of TGF-β can restore cytotoxic gene expression in CTLs, recover their anti-tumor activity, reestablish metabolic balance, and remodel the TME to enhance the efficacy of cancer immunotherapy (73). For instance, a BsAb designed for the dual blockade of PD-L1 and TGF-β has showed therapeutic potential (74). Furthermore, 4T-Trap (CD4xTGF-β Trap)-a bispecific receptor decoy that anchors the TGF-β-type II receptor to CD4(+) T cells- selectively inhibits TGF-β signaling in tumor-draining lymph nodes, reorganizes tumor vasculature, and induces cancer cell death, implicating that blocking TGF-β signaling can remodel the TME to prevent cancer progression (75). Consequently, combining EAT therapy with TGF-β blockade can further amplify therapeutic responses by alleviating immunosuppression and revitalizing the intratumoral immune response.

Targeting the VEGF pathway is another promising approach to address the hypoxic and acidic TME, which enhances the therapeutic efficacy of EAT therapy. VEGF drives abnormal neovasculature and intratumoral hypoxia, contributing to an immune-hostile TME. The VEGF-A/VEGFR signaling pathway downregulates adhesion and chemotactic signals on the tumor endothelium, hindering the trafficking and infiltration of CTLs (76), and promotes tumor-induced Treg proliferation (77). While the monoclonal antibodies and tyrosine kinase inhibitors targeting this pathway have shown limited efficacy as a single treatment (78), combined therapies of bevacizumab and T cell immunotherapies have demonstrated a synergy by improving T cell migration and their activity (79–81). When VEGF targeting strategies [bevacizumab or anti-VEGFR2 antibody (DC101)] were combined with EAT therapy, EATs’ infiltration into tumor was much improved, accelerating tumor shrinkage without significant cytokine release and inducing durable responses. This combination induced formation of high endothelial venules (HEVs), specialized post-capillary venules that facilitate lymphocyte trafficking. These HEVs were associated with increased density and broader distribution of CD8(+) TILs (82, 83). Anti-angiogenic therapy promotes transdifferentiation of post-capillary venules into inflamed HEVs via lymphotoxin (LT)-LTβR signaling (84), and tumor HEVs create permissive niches for CTL infiltration and expansion (85). Consistently, anti-VEGF therapies increased HEV formation in the TME and improved EAT dispersion, effectively overcoming T cell exclusion (82). Interestingly, tumor-HEV induction did not follow MDSC or TAM depletion, unlike anti-VEGF therapies (82).

Recently, VEGFxPD-1 dual-targeting BsAb, ivonescimab, has demonstrated superior survival outcomes in patients with advanced non-small cell lung cancer (NSCLC) by simultaneously normalizing tumor vasculature and alleviating immune checkpoints (86). Integrating such constructs into the EAT platform (including multi-EAT) may offer a synergistic opportunity. Unlike traditional CD3-based arming, VEGF×PD-1 constructs should utilize the upregulated PD-1 receptors on activated, ex vivo-expanded T cells as a docking site for arming. While PD-1 is often associated with T cell exhaustion in the context of chronic tumor exposure, it is also rapidly upregulated as a physiological activation marker following ex vivo costimulation (56, 87). By harvesting T cells at their peak activation (days 7–10 of expansion), the transiently high expression of PD-1 can be leveraged to anchor VEGF×PD-1 BsAb. This strategy may provide a dual advantage: the PD-1 blockade shield EATs from TME-induced exhaustion, while the anti-VEGF domain normalizes tumor vasculature to ensure T-cell infiltration. Although subsequent PD-1 downregulation might lead to the shedding or internalization of the anchored BsAb, the persistent PD-1 expression on exhausted T cells could potentially serve as a secondary anchor, maintaining the therapeutic engagement within the immunosuppressive TME. These multi-mechanistic approaches would address the physical barriers of the TME while maintaining peak metabolic fitness of the effector T cells, offering a potent synergy.

## 6 Unlocking the potential of EAT therapy

### 6.1 Simultaneous multi-antigen targeting strategy

The most common reason for treatment failure after targeted immunotherapies is tumor heterogeneity and mechanisms of antigen escape, such as target antigen loss or downregulation. Most human cancers exhibit heterogeneous antigen expression, and the tumor-associated antigens (TAAs) continue to evolve under selective immune pressure following targeted therapies, causing treatment resistance (88). Despite its remarkable efficacy, antigen loss leads to disease relapse or treatment failure in approximately 30%–70% of patients receiving CAR-T therapy (7). Multi-antigen targeting approaches have the potential to tackle these hurdles: Tandem CAR-T (two scFvs on a single stalk) targeting HER2/IL-13Rα2, EGFRvIII/IL-13Rα2, or CD19/CD20 and dual CAR-T (two CARs from a bicistronic construct) targeting CD19/CD37 effectively mitigated antigen escape and showed superior antitumor activity compared with monospecific CAR-T (mono-CAR-T) in heterogeneous antigen-expressing tumor models (89–92). The EAT platform facilitates a ‘plug-and-play’ approach, allowing multiple BsAbs targeting distinct antigens to be seamlessly assembled onto T cells without complex genetic engineering. While each BsAb must be independently produced under strict GMP manufacturing and quality control protocols, these multiple BsAbs can be easily assembled onto T cells to achieve the intended multi-specificity. This ‘plug-and-play’ capability allows for the rapid adaptation of the therapy to the heterogeneous antigenic landscapes of solid tumors, providing a flexible alternative to the fixed genetic architectures of traditional CAR-T products (93). Given the minimal requirement of BsAb (only 500–5000 molecules) per T cell for antitumor activity (24), multiple BsAbs can be installed on each T cell before the maximum capacity is reached (50). While the *in vitro* cytotoxicity of multi-EATs was similar to those of mono-specific EATs against each individual target, multi-EATs elicited more potent and durable antitumor effects against heterogeneous tumors (24, 93). When evaluating efficacy among different arming strategies, dual-EATs (two distinct BsAbs on a single T cell) outperformed pooled mono-EATs and tandem EATs (T cells armed with a single trispecific Ab comprising two distinct scFvs linked to anti-CD3 scFvs) in treating heterogeneous tumors (93). This mirrors findings in the CAR-T studies, where bicistronic dual-CAR-T demonstrate superior efficacy against heterogeneous tumors compared to pooled or tandem versions (94), supporting the idea that expressing two distinct binding moieties on a single cell mechanistically enhances the interactions between effector and target cells.

Current multi-antigen strategies utilize AND-gated logic to enhance specificity or OR-gated logic to counter tumor heterogeneity and antigen escape. While AND-gated CAR-T minimize ‘on-target off-tumor’ toxicities by requiring dual-antigen recognition, OR-gated CAR-T broaden therapeutic reach at the cost of increased risk of systemic toxicity (95). The multi-EATs primarily utilizes an OR-gated logic to target a broad spectrum of antigens (93). However, multi-EATs offer a distinct safety profile differentiated from traditional OR-gated CAR-T by leveraging reduced individual antigen avidity and a finite lifespan. Reducing target antigen affinity to a certain threshold (Kd <10^−8^ M) could decrease toxicities without affecting anti-tumor efficacy (40, 96), suggesting that avidity optimization is an effective strategy to reduce unwanted target recognition (97, 98). Additionally, cytokine removal after arming further decrease the risk of systemic toxicities potentially induced by multi-EATs (99).

### 6.2 Selective and simultaneous targeting of cancer stem cell using the EAT platform

By leveraging these advantages of EAT—safety and the easy assembly of multiple BsAbs on a T cell—precision medicine can be tailored to the unique tumor characteristics of each patient. One approach is to target CSC and cancer surface antigens simultaneously, which may enhance the chance of achieving a cure. CSC constitutes the most aggressive subpopulation within the tumor mass, characterized by their invasive properties, metastatic potential, and high expression of drug efflux pumps that drive treatment resistance, however, selective targeting of CSC is still an unmet need in cancer treatment (100). CSC downregulates the expression of the MHC class I molecules to elude CTL recognition, thus the BsAb- or CAR-T-based approach may be an optimal strategy to target them (27, 101). A substantial number of CSC-directed strategies using BsAb or CAR-T are actively being investigated. These include anti-CD44v6 CAR-T targeting AML and MM (102), anti-CD166 CAR-T for osteosarcoma stem cells (103), anti-CD133 CAR-T for gastric, pancreatic, and hepatic CSCs (104, 105), and AC133 (epitope of CD133)-targeting T-BsAbs for GBM CSCs (106). Anti-IL1R-L1 (receptor for cytokine IL-33) T-BsAb targeting both leukemia stem cells (LSCs) and IL-1RL1(+) immune suppressor cells effectively decreased leukemic burden, reversed TME-mediated immune tolerance, and restored anti-leukemia immunity, leading to improved survival (107, 108). By harnessing the multi-EAT strategy, multiple TAAs and CSCs can be simultaneously targeted, and these ‘multi-potent’ EATs are expected to enable a comprehensive approach, leading to more effective tumor elimination while preventing recurrence.

## 7 Critical considerations for the clinical translation of EAT

While EATs offer a modular alternative to BsAb and CAR-T therapies, they possess significant operational requirements. The clinical translation of EATs necessitates rigorous GMP-grade T cell expansion, the procurement of GMP-grade BsAbs, and logistical coordination for repeated dosing and additional cytokine support. Consequently, EATs represent a strategic middle ground among the T cell redirection modalities. They face inherent scalability limitations compared to mass-produced recombinant BsAbs, yet they offer a distinct logistical advantage over CAR-T by bypassing viral vector transduction and utilizing ‘off-the-shelf’ antibodies. As BsAb engineering evolves toward high-potency recombinant formats, such as IgG-[L]-scFv, and manufacturing transitions toward automated closed-system platforms, EAT therapy is expected to become more scalable and affordable. Ultimately, transitioning to allogeneic T cells will enable the development of truly ‘off-the-shelf’ EAT therapy. By integrating with strategies targeting the TME or VEGF pathways, these advancements will reduce treatment burden and facilitate broader clinical implementation.

One of the main limitations in the current landscape is the absence of clinical trials directly comparing identical BsAb constructs as monotherapies versus as EATs. As EAT therapy remains in preclinical and early clinical development, this lack of head-to-head data precludes definitive conclusions regarding their relative therapeutic superiority. Nevertheless, EATs offer a compelling alternative to address one of the major limitations of BsAb therapy. By providing pre-activated, ex vivo expanded T cells armed with BsAbs, EATs may overcome the quantitative and functional insufficiency of endogenous T cells observed in patients with advanced cancer. Furthermore, the removal of unbound BsAbs and released cytokines before infusion may reduce systemic toxicities and improve tolerability. Thus, although comparative clinical validation is still needed, EATs offer a rational platform to enhance T-cell redirection in solid tumors by combining antibody-mediated tumor targeting with a defined effector-cell product.

## 8 Conclusion

Beyond the duopoly, EATs should not be viewed merely as an alternative, but as a distinct and potent modality that offers unique advantages in flexibility and tumor infiltration. While CAR-T and BsAbs represent the current pillars of T-cell redirection therapy, inherent limitations persist—particularly regarding the dynamic and evolving nature of solid tumors. Arguably, more controllable and cost-effective modalities are required to bridge the gap between these established platforms. By combining efficient tumor infiltration with modular adaptability to heterogeneous antigens, EATs offer a unique facile strategy to overcome the biological and logistic hurdles presented by solid tumors.

To further enhance efficacy, combination strategies are available to target the immunosuppressive TME, including tumor-infiltrating MDSCs, TAMs, Tregs, and immunosuppressive cytokines, as well as the VEGF signaling pathway. Furthermore, harnessing high-throughput target discovery alongside the standardization of protocols for multi-EAT production holds the potential to mitigate immune escape to significantly improve therapeutic outcomes in complex solid tumors. Beyond conventional αβ T cells, other effectors such as NKT cells, mucosal-associated invariant T (MAIT) cells, and γδ T cells provide further opportunities for developing “off-the-shelf” EAT therapies. Ultimately, overcoming the barriers of the solid tumor microenvironment requires broadening our perspective beyond established platforms. Our results underscore that in the evolving landscape of immunotherapy, every specialized tool—including EATs—could play a crucial and non-redundant role in achieving curative outcomes.

## Glossary

AICD: activation-induced cell death
APC: antigen presenting cell
BBB: blood-brain barrier
BiTE: bispecific T cell engager
BsAb: bispecific antibody
CAR-T: chimeric antigen receptor T cells
CRS: cytokine release syndrome
CSC: cancer stem cell
CSF1R: colony-stimulating factor 1 receptor
CTL: cytotoxic T lymphocyte
CTLA-4: cytotoxic T lymphocyte-associated antigen 4
EAT: ex vivo armed T cell
EC50: half maximal effective concentration
EGFR: epidermal growth factor receptor
EpCAM: epithelial cell adhesion molecule
FcRn: neonatal fragment crystallizable receptor
FcγR: Fc gamma receptor
GBM: glioblastoma multiforme
HCT: hematopoietic stem cell transplantation
HER2: human epidermal growth factor receptor
HER2/neu: human epidermal growth factor receptor 2/neuroblastoma
HEV: high endothelial venule
HLA: human leukocyte antigens
ICANS: immune effector cell-associated neurotoxicity syndrome
ICI: immune checkpoint inhibitor
IDO: indoleamine 2,3-dioxygenase
IFN-γ: interferon-γ
IL1RL1: interleukin-1 receptor-like 1
MDSC: myeloid-derived suppressor cells
MET: mesenchymal-epithelial transition factor
MHC: major histocompatibility complex
NB: neuroblastoma
NHL: non-Hodgkin lymphoma
NK cell: natural killer cell
NKT cell: natural killer T cell
PD-1: programmed cell death protein-1
PD-L1: programmed cell death ligand 1
scFv: single chain fragment variable
TAA: tumor-associated antigen
TAM: tumor-associated macrophage
TGF-β: transforming growth factor-β
TME: tumor microenvironment
TNF-α: tumor necrosis factor-α
Treg: regulatory T cell
VEGF: vascular endothelial growth factor
Wnt: wingless
